# Analysis of Kojic Acid Derivatives as Competitive Inhibitors of Tyrosinase: A Molecular Modeling Approach

**DOI:** 10.3390/molecules26102875

**Published:** 2021-05-12

**Authors:** Richelly Cardoso, Renan Valente, Clauber Henrique Souza da Costa, João Lidio da S. Gonçalves Vianez, Kauê Santana da Costa, Fábio Alberto de Molfetta, Cláudio Nahum Alves

**Affiliations:** 1 Laboratório de Modelagem Molecular, Instituto de Ciências Exatas e Naturais, Universidade Federal do Pará–UFPA, Guamá, Belém-PA 66075-10, Brazil; richellycardoso10@yahoo.com.br (R.C.); fabioam@ufpa.br (F.A.d.M.); 2 Laboratório de Planejamento e Desenvolvimento de Fármacos, Instituto de Ciências Exatas e Naturais, Universidade Federal do Pará–UFPA, Guamá, Belém-PA 66075-10, Brazil; cr.henriquecosta@gmail.com; 3 Laboratório de Sistemas Moleculares Complexos, Instituto de Ciências Exatas e Naturais, Universidade Federal do Pará–UFPA, Guamá, Belém-PA 66075-10, Brazil; rdapenhavalente@gmail.com; 4 Center of Technological Innovation, Evandro Chagas Institute, Ministry of Health, Ananindeua-PA 67030-000, Brazil; vianez.iec@gmail.com; 5 Universidade Federal do Oeste do Pará, Instituto de Biodiversidade, Santarém-PA 68035-110, Brazil

**Keywords:** skin cancer, melanogenesis, tyrosinase, kojic acid derivatives, molecular docking, molecular dynamics

## Abstract

Tyrosinases belong to the functional copper-containing proteins family, and their structure contains two copper atoms, in the active site, which are coordinated by three histidine residues. The biosynthesis of melanin in melanocytes has two stages depending on the actions of the natural substrates L-DOPA and L-tyrosine. The dysregulation of tyrosinase is involved in skin cancer initiation. In the present study, using molecular modeling tools, we analyzed the inhibition activity of tyrosinase activity using kojic acid (KA) derivatives designed from aromatic aldehydes and malononitrile. All derivatives showed conformational affinity to the enzyme active site, and a favorable distance to chelate the copper ion, which is essential for enzyme function. Molecular dynamics simulations revealed that the derivatives formed promising complexes, presenting stable conformations with deviations between 0.2 and 0.35 Å. In addition, the investigated KA derivatives showed favorable binding free energies. The most stable KA derivatives showed the following binding free energies: −17.65 kcal mol^−1^ (D6), −18.07 kcal mol^−1^ (D2), −18.13 (D5) kcal mol^−1^, and −10.31 kcal mol^−1^ (D4). Our results suggest that these derivatives could be potent competitive inhibitors of the natural substrates of L-DOPA (−12.84 kcal mol^−1^) and L-tyrosine (−9.04 kcal mol^−1^) in melanogenesis.

## 1. Introduction

Skin cancer is a global public health problem, and approximately 132,000 new cases of malignant melanoma are registered each year [1,2]. Melanomas can be caused by the dysregulation of the enzymatic activity of tyrosinase, an enzyme responsible for the biosynthesis of melanin. Melanogenesis corresponds to the biochemical process involved with the production and distribution of melanin pigment [3,4], and it is modulated by genetic, hormonal, and environmental factors, such as exposure to ultraviolet rays and abnormal hormonal production [3,5,6,7]. 

**Figure 1:**
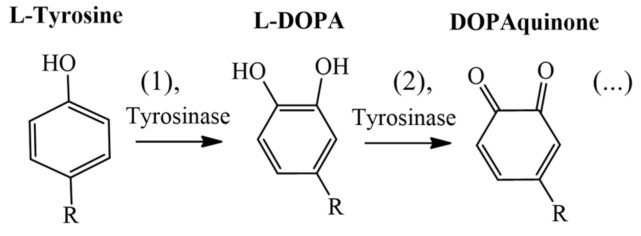
Catalytic stages (1 and 2) performed by tyrosinase in the biosynthetic pathway of melanin.

Tyrosinases belong to the functional copper-containing proteins family, and their structure contains two copper atoms in the active site, which are coordinated by three histidines [8,9,10]. The biosynthesis of melanin in melanocytes has two stages depending on the actions of the natural substrates L-DOPA and L-tyrosine. Figure 1 shows the main stages of melanin synthesis in melanocytes, which include the formation stages of L-DOPA and DOPAquinone, by the catalytic action of tyrosinase. The synthesis begins when the tyrosinase hydroxylates the L-tyrosine (1) producing L-DOPA, which is oxidized (2), producing DOPAquinone. Then, successive chemical reactions are triggered until melanin is completely formed [11,12]. Due to its determinant role in the biosynthetic pathway of melanin, tyrosinase has been widely investigated as an interesting molecular target with applications in the pharmaceutical and cosmetic industries [13,14,15,16,17].

Kojic acid (KA) is a natural compound widely studied as a competitive inhibitor of tyrosinase [18,19]. KA prevents the formation of melanin in human melanocytes due to the reversible inhibition of tyrosinase, but it has some side effects, such as skin irritability and instability [20]. In contrast, some changes in its molecular structure have been shown to circumvent these effects and improve the efficiency of its inhibitory activity [19,21].

**Figure 2:**
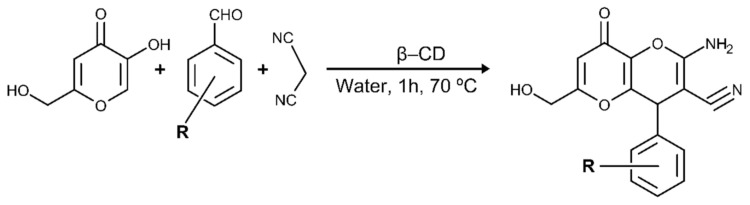
Synthesis of KA derivatives obtained from malononitrile, aldehyde, and KA and catalyzed by β-CD previously reported by Kataev et al. (2016) [24].

Different experimental studies using enzymatic inhibition assays have demonstrated that some KA derivatives act as inhibitors of tyrosinase activity [19,21,22,23]. Furthermore, some synthetic methods based on the principles of green chemistry have been shown to be efficient in reducing or eliminating the production of toxic substances during the synthesis of bioinspired compounds [19,24]. The synthesis of KA derivatives obtained from malononitrile, aromatic aldehyde, and KA catalyzed by β-cyclodextrin (β-CD) is temperature-dependent catalysis occurring at 70 °C in the presence of water [25]. Figure 2 shows the chemical reaction involved in the green synthesis of these derivatives. 

These derivatives interact with tyrosinase by a competitive process similar to KA [25], inhibiting the interaction of the natural substrate L-tyrosine, thus preventing the formation of the successive L-DOPA. These competitive inhibitors can also act in the second stage, preventing the formation of DOPAquinone, and consequently, preventing melanin biosynthesis [26]. In the present study, using a molecular modeling approach, we described the selectivity and affinity of KA derivatives against tyrosinase structure and investigated their drug-like properties. Our results shed light on the molecular mechanism of action of these compounds and indicate that their structures could act as potential inhibitors of tyrosinase activity.

## 2. Material and Methods

### 2.1. KA Derivatives Based on Aromatic Aldehydes and Malononitrile

The fourteen KA derivatives (named D1 to D14, see IUPAC names in Table S1) were initially designed in the Marvin Sketch (version 18.2) program [27]. Then, these structures were converted to 3D representation, and their geometries were optimized by the semi-empirical method PM7 [28] in the Molecular Orbital PACkage (MOPAC) program [29], to obtain the lower energy conformations. All KA derivatives designed contain the phenyl group in their structures. The D2, D4, D5, D6, D7, D10, D11, and D14 derivatives contain an oxygen atom attached to the phenyl group. The derivatives D1, D3, D8, D9, D12, and D13 also contain the halogens Cl, F, Br in the *ortho, meta,* and *para* positions of the aromatic ring. The derivatives D4 and D5 are phenolic compounds, and the D2 and D6 contain ether groups at the R position. 

### 2.2. Evaluation of Drug-like Properties

To analyze the drug-like properties of the selected KA derivatives, we investigated their physicochemical and structural properties using the InstantJChem program. All molecular properties were selected according to the following chemical rules applied to analyze drug-like compounds: Lipinski’s Rule of 5’ (RO5) [30], Veber [31], and Muegge [32]. 

### 2.3. Molecular Docking

The molecular docking analyzes were performed using the CSD Gold program (version 5.5) [33], and the molecular interactions were analyzed in the PoseView [34]. CSD Gold uses a genetic algorithm to predict the binding modes of the ligands in the receptor binding site, using the principles of the biological evolution [35] where a chromosome population is responsible for the configuration of the ligand poses, such as dihedral angles and intramolecular bonds.

The tyrosinase structure complexed with the KA was obtained from the RCSB Protein Data Bank using the accession code: 5I38 (resolution: 2.6 Å, chain: A) [9]. This structure has two homologous chains (A and B), each one containing 286 residues, two copper metals (named here as, Cu-A and Cu-B), and the KA, as a competitive inhibitor, complexed to the active site. Initially, all water molecules were removed and the hydrogens were added to the tyrosinase structure. The docking grid with a cavity radius of 12.19 Å was positioned to the same spatial coordinates of KA complexed to the crystallographic binding site (Cartesian coordinates of the center of the cavity: x = 1.93, y = 101.58, and z = 25.27). First, to validate our docking protocol, we performed a redocking simulation of KA complexed with the crystallographic structure of tyrosinase (PDB code: 5I38), and the protocol that reached poses with root mean square deviation value (RMSD) ≤ 1 Å in relation to the experimental structure was selected to perform the docking simulations. Then, fourteen KA derivatives were docked against the tyrosinase binding site using the following parameters: number of runs = 10, population size = 100, crossing over rate = 95, mutation frequency = 95 (Table S2). These fourteen KA derivatives were docked using the GoldScore scoring function that is derived empirically from a set of 82 protein-ligand complexes [36].

The docking simulation was applied as a pre-filtering method to select the most promising inhibitors of tyrosinase. Finally, the best poses obtained from the KA derivatives were selected based on their docking scores. Considering that copper chelation is essential to inhibit the tyrosinase activity, we also performed a visual inspection regarding the formation of interatomic distances of the KA moiety from the derivatives with the copper ion. Thus, to select the best docking poses for further analyses, we assumed a cutoff of ≤4Å for the interatomic distance of the KA moiety with the copper ion. 

### 2.4. Molecular Dynamics (MD) Simulation 

**Figure 3:**
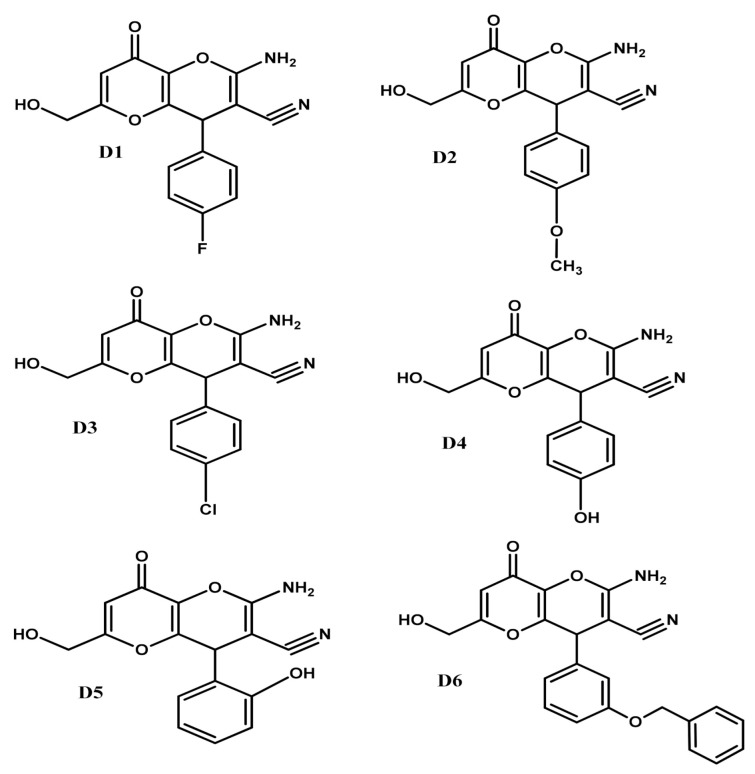
Molecular structure of the six KA derivatives (D1 to D6) analyzed during the MD simulations.

MD simulations were performed in Q package [37,38] to analyze the selectivity and stability of the substrates (L-DOPA and L-tyrosine) and six selected KA derivatives (named D1 to D6, see Figure 3) complexed with tyrosinase structure. The MD simulation consisted of four main stages: preparation, minimization, heating-balance, and production. The copper ions were treated using the nonbonded dummy atoms model to redistribute the atomic charges and reduce the excessive repulsion in the metallic region [38,39]. Initially, dummy atoms were added around the copper atoms, using the UCSF Chimera [40]. The protonation states of the ionizable residues were analyzed in the PROPKA server [41], using pH 6.8, which is within the optimum range for the enzyme [42]. In the preparation stage, the OPLSAA force field was applied to treat the systems [43], then it was solvated in a 20 Å spherical water-box using the TIP3P model and the temperature (300 K) and pressure (1 bar) control were maintained with a Berendsen thermostat and pressure algorithm of Q program, respectively [44]. Then, the systems were neutralized by the Q-prep program. In the minimization and heating stages, the temperatures of the complexes were increased from 0 to 300 K and were balanced at 50 ps. We started to save the frames at 250 ps, with a time interval of 1.0 fs, then the frames were saved in a periodic interval of 25 fs. For long-range electrostatic interactions, we applied the expansion approximation of the local reaction field [45]. Spatial restraints were applied for the copper and the histidine atoms with a force of 6 kcal/mol^−1^Å^−2^ and 8 kcal/mol^−1^Å^−2^, respectively. An energy constraint of 0.1 kcal/mol^−1^Å^−2^ was also applied to maintain the inhibitor at the center of the solvation sphere during all stages of the MD simulation. The root means square deviation (RMSD) values were computed using the R program and BIO3D package [46].

### 2.5. Binding Free Energy Calculations

To analyze the binding affinity of the ligands complexed to the tyrosinase active site, we performed binding free energy calculations using the linear interaction energy (LIE) method [47]. LIE is described according to Equation (1).

(1) $$∆G_{LIE}^{bind}=\;\alpha \;(\langle V_{\left(l-s\right)}^{vdW}\rangle {\;}_{bind}-\;\langle V_{\left(l-s\right)}^{vdW}\rangle {\;}_{free})+\;\beta \;(\langle V_{\left(l-s\right)}^{el}\rangle {\;}_{bind}-\beta \;\langle V_{\left(l-s\right)}^{el}\rangle {\;}_{free})$$

where 〈〉 denotes averages of the van der Waals (vdW) and electrostatic (el) interaction energies, the term (l − s) corresponds to the ligand surrounding energies, α is the empiric correction factor of Van der Waals interactions; and β is the correction factor of electrostatics interactions. 

Here, we selected 10,000 frames (5ns) from the MD trajectory to compute the energies using the α and β empirical correction parameters (α value = 0.181 and the β values = 0.33 and 0.37) [44]. In the present study, we combined MD simulations performed in Q package [38,48] with binding free energy calculations performed with the LIE method [47,49,50]. This computational protocol has been widely applied by our research group for computer-aided drug design due to its satisfactory approach to describe different molecular biosystems [17,51].

## 3. Results and Discussion

Melanin biosynthesis plays an important role in protecting the skin cells against damage, and non-melanin production consists of an abnormal condition, associated with clinical manifestations, such as vitiligo and albinism [9]. Considering that natural compounds remain one of the most interesting sources for the design and synthesis of new bioactive compounds with pharmaceutical and cosmetic applications [52,53,54], here using computational tools, we analyzed the drug-like properties, as well as the selectivity and affinity against the tyrosinase binding site of fourteen KA derivatives, which could be synthesized through green chemistry routes.

### 3.1. Analysis of Selectivity of the KA Derivatives to the Tyrosinase Binding Site

Different studies have reported that KA derivatives act as inhibitors of tyrosinase [19,21,22,23]. Initially, we investigated the selectivity of fourteen KA derivatives against the tyrosinase binding pocket using molecular docking, a computational tool widely applied in structure-based virtual screening approaches [55,56,57,58,59,60,61,62,63]. First, re-docking simulations using the crystallographic structure of KA were performed in the CSD Gold [33] program to validate our docking protocol. The redocking simulations obtained an RMSD value equal to 0.125 Å when compared with the reference crystallographic structure (PDB: 5I38) [9]. The small RMSD deviation values obtained from the redocking simulation validated our docking protocol. The interatomic distances between the KA and copper ion indicated by crystallographic structure were 4.89 Å (Cu-A) and 5.51 Å (Cu-B), while re-docking obtained 4.79 Å (Cu-A) and 5.53 Å (Cu-B). The molecular docking simulations demonstrated that all KA derivatives showed a better affinity to the tyrosinase binding pocket than the KA (Table 1). 

The non-covalent interactions of the ligand-receptor complexes were identified using the PoseView program. The *pose* obtained from molecular redocking of KA showed similar interactions from those found in the crystallographic structure (PDB ID: 5I38) [9]. The interactions included Pi-donor H-bond and π-π stacked (His208), conventional H-bonds (Val217), and carbon-hydrogen bond/π-sigma (Val218). 

### 3.2. Analysis of the Interaction of KA Derivatives with Tyrosinase Binding Pocket

**Figure 4:**
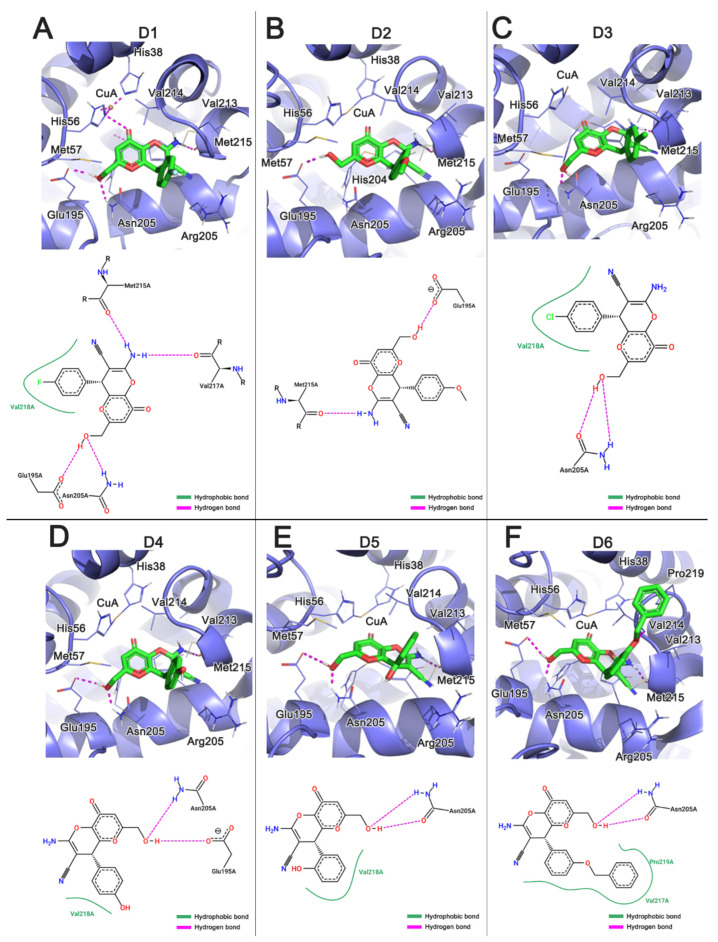
Intermolecular interactions obtained from molecular docking of KA and its derivatives against the tyrosinase binding pocket. (**A**) Derivative D1; (**B**) D2; (**C**) D3; (**D**) D4; (**E**) D5; (**F**) D6.

The analyzed KA derivatives showed binding energies similar to the natural substrates and KA, and D6 showed the most favorable binding affinity to the tyrosinase active site. Figure 4A–F shows the main interactions formed between the KA derivatives with the tyrosinase active site. The interactions of the derivatives with the residues Glu195, Met215, Val217, Val218, His208, and Asn205 proved to be relevant for receptor recognition. It is important to note that three residues His208, Val217, and Val218 were previously described forming interactions with the KA structure. In the present study, we identified new interactions between the KA derivatives and the residues Glu195, Met215, and Asn205 due to their substituted phenyl group. The identified residues have been reported to be important for stabilizing the interactions of the derivatives with the active site [12]. Furthermore, the formation of hydrogen bonds with the residues Met215, Asn205, and Glu195 have been shown to be relevant for the molecular recognition and stabilization of the interactions with the binding pocket [64,65].

The carbonyl group of Met215 interacted with the amino group of the derivatives, while the amino group of Asn205 interacted with the oxygen of these derivatives. The hydroxyl of the derivatives interacted with the residue Asn205 and Glu195, respectively. The residue Val218 formed hydrophobic interactions with all analyzed derivatives through the aromatic ring. Similarly, Pro219 also formed hydrophobic contacts with the D6 derivative. It is important to note that the derivative D6 satisfactorily occupied the tyrosinase binding pocket when compared with the KA and other analyzed derivatives. Interestingly, we noted that D6 also impaired the entrance of the catalytic site, leading to the inhibition of the entrance of the substrate L-tyrosine. 

The molecular docking results showed that the KA derivatives [65] have a high conformational selectivity to the tyrosinase when compared with kojic acid, mainly due to the presence of a substituted phenyl group, which formed new interactions with the residues of the binding pocket. All derivatives showed favorable interactions that are consistent with the chelation of copper ions (Cu-A and Cu-B) (Table S3). Thus, we conjecture that these derivatives have the potential to mimic the binding mode of the natural substrates L-DOPA and L-tyrosine in melanin biosynthesis. 

### 3.3. Analysis of Interactions between the Histidine Residues and the Copper Ions 

Despite the relevance of metals for catalysis and structural stability of enzymes, the MD simulations of metal–enzyme systems are a challenge in molecular modeling [55,66,67]. The dummy atoms model [37] represents an advance in the description of these enzymatic systems, since this model captures the structural and electrostatic effects through the introduction of fictitious atoms around the metal ion, redistributing the charges and reducing the excessive repulsion in the metal region. Therefore, instead of a simple sphere with a point charge, the dummy model redistributes the metallic charge to other atoms with partial charges [38,68]. This computational strategy has been successfully applied in different metal–enzyme systems to perform MD simulations and to predict protein structures [69,70,71,72].

In the active site of tyrosinase structures, the histidine residues play an important role in stabilizing the copper ions. In the present study, the chemical structure of these metals was elucidated using the dummy atom methodology [38,39], which consists of redistributing the copper point charge to six fictitious atoms with partial charges. Interestingly, we noted that all systems showed stable interactions with the histidine, with distance variation around 2.0 Å (Table S4), demonstrating that the application of dummy atoms coherently described the organometallic structure of tyrosinase, allowing the interpretation of the chelation of metals by KA and its derivatives.

### 3.4. Analysis of KA Derivatives Complexed with Tyrosinase over MD Simulations

**Figure 5:**
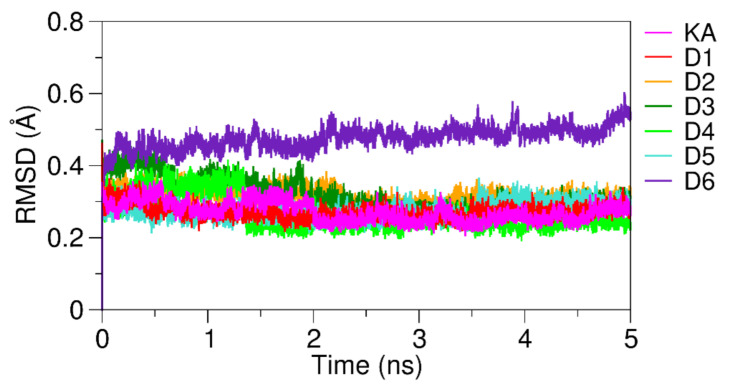
RMSD plots of the tyrosinase structure complexed with KA and its derivatives.

To describe the selectivity and affinity of KA and its derivatives with the tyrosinase active site, as well as the enzyme interactions with copper metals, MD simulations were performed for all analyzed protein–ligand systems with a total time of 5 ns using the Q package. Figure 5 show the RMSD plots obtained over 5 ns of MD simulation. RMSD is a statistical measure of the spatial variation of the complex concerning the coordinates of the reference structure [46,68,73]. We analyzed the RMSD plots over the MD simulation to investigate the stability of the ligand–receptor complexes. Satisfactory, the RMSD values for the KA and its derivatives were obtained between 0.2 to 0.35 Å, demonstrating that the tyrosinase–ligand complexes reached a stable conformation over the MD trajectory (Figure S1). The D6 complexed with tyrosinase presented a small deviation of RMSD values when compared with other derivatives systems stabilizing at 0.5 Å. The natural substrates L-DOPA and L-tyrosine also showed a stable interaction at 0.2 Å (Figure S2).

### 3.5. Analysis of Chelation of Copper Ions

Copper chelation is the main mechanism of enzymatic inhibition of tyrosinases [74]. The interactions and conformational stability of KA and its derivatives with the copper ions of tyrosinase were investigated by MD simulations. All systems showed stability consistent with the ion chelation with a mean distance of 5.63 Å for Cu-A and 3.51 Å for Cu-B (Table 2). It is important to note that all derivatives interacted at a shorter distance with the Cu-A ion. In contrast, KA interacted more closely with Cu-B compared to the analyzed derivatives. Our computational results indicate that these investigated KA derivatives are potential competitive inhibitors of the natural substrates L-DOPA and L-tyrosine due to their strong affinity to the active site of tyrosinase and its proximation to copper, which favors the metal chelation. 

### 3.6. Binding Affinity of KA Derivatives to the Tyrosinase Active Site

The conformational stability of the six KA derivatives complexed to the tyrosinase active site was analyzed by MD simulations. Then, binding free energy calculations using the LIE method were performed at an interval of 5 ns to analyze the binding affinity of the complexes. It is important to highlight that the LIE method does not require a long MD simulation, thus we selected a short interval of the trajectory to analyze the binding free energy of the ligand–receptor complexes [75]. Table 3 shows the energetic contributions of the KA and its investigated derivatives complexed with tyrosinase.

The terms $V_{l-s}^{\mathrm{vdW}}\;\mathrm{bind}$ and $V_{l-s}^{\mathrm{vdW}}\;\mathrm{free}$ represent unbound interactions of the complex and the solvated ligand, respectively; and the terms $V_{l-s}^{el}\;\mathrm{comp}$ and $V_{l-s}^{el}\;\mathrm{free}$ correspond to the interactions of the electrostatic potentials of the complex and the solvated ligand, respectively. The Van der Waals interactions, which represent the unbound interactions between the derivatives and the binding pocket, showed the lowest energetic contributions. In contrast, the electrostatic contributions showed higher contributions, due to the presence of the hydroxyl group of the derivatives interacting with some residues of the catalytic site. The D2, D5, and D6 derivatives showed the following binding free energies: −18.07, −18.13, and −17.65 kcal mol^−1^, respectively. These results demonstrated that these derivatives formed stable complexes with tyrosinase during MD simulation when compared with KA, which showed a binding free energy equal to −5.00 kcal mol^−1^. According to our molecular analysis of the drug-like properties of the analyzed compounds, the six KA derivatives (D1 to D6) were approved by Lipinski, Veber, and Muegge molecular rules (Table S5), which indicate that their structures and physicochemical properties are compatible with commercial drugs approved for human use and these compounds exhibit pharmacokinetic properties compatible with gastrointestinal absorption.

Regarding the binding affinity of the investigated compounds, the KA showed binding energy to the tyrosinase binding site equal to −5.00 kcal mol^−1^, and the D1 and D3 derivatives showed similar energies with −4.42 kcal mol^−1^ and −5.09 kcal mol^−1^, respectively. Similarly, the D4 derivative showed binding energy similar to the natural substrates L-DOPA and L-tyrosine (see energy decomposition analysis in Figures S3 and S4). In contrast, the derivatives D6, D5, and D2 showed a satisfactory affinity to the tyrosinase active site, which is consistent with the inhibition of the enzymatic activity. It is important to highlight that our computational analysis does not aim to identify the inhibitory concentrations of the investigated compounds, which is one of the main factors involved in the inhibition of enzymes. Thus, further experimental evaluations will be required to test their inhibition against tyrosinase.

### 3.7. Analysis of Pairwise Energy Decomposition

The pairwise energy decomposition indicates the energetic contributions (electrostatic and Van der Waals) of each residue involved in the stabilization of the ligands with the enzyme binding pocket. This computational method has been applied to describe protein–protein [50,70,76,77] and ligand–protein interactions [55,78,79,80,81]. 

**Figure 6:**
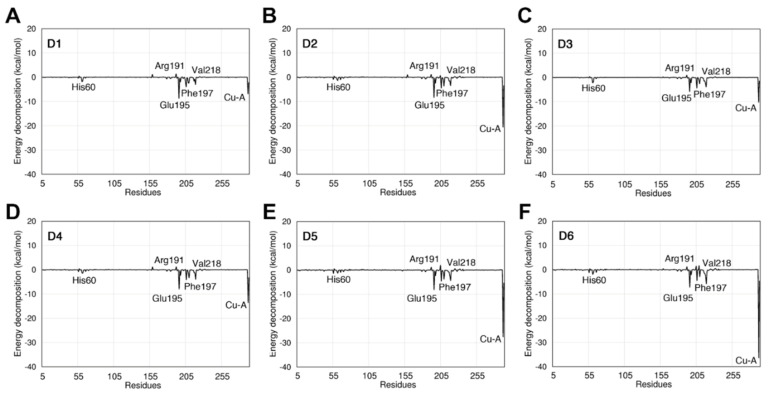
Pairwise energy decomposition analysis of the residues located at the tyrosinase binding pocket complexed with the KA derivatives, (**A**) Derivative D1; (**B**) D2; (**C**) D3; (**D**) D4; (**E**) D5; (**F**) D6.

The analysis of pairwise energy decomposition of residue interactions was performed for KA (Figure S5) and its derivatives (Figure 6) complexed with the tyrosinase binding site. Our analyses demonstrated a similar energetic profile when analyzing the electrostatic terms of the energy decomposition and Van der Waals. The analysis of the contribution of the individual residues showed that the residues Leu194, Asn205, Val218, and Leu290 formed favorable interactions with KA derivatives. These residues are near the active site and they contribute to the stability of the KA derivatives. The derivatives formed favorable interactions with residues located at the tyrosinase binding site, and new interactions when compared with the KA, thus indicating their higher affinity with the binding pocket. The natural substrates L-DOPA and L-tyrosine showed a similar decomposition profile of the analyzed inhibitors (KA and its derivatives). However, the electrostatic component showed a relevant contribution to these inhibitors. The Glu195 residue showed a greater energy contribution in the interaction with the inhibitors showing a value of −1.07 kcalmol^−1^ when in complex with kojic acid. We also identified that the residues His60, Arg191, Phe197, and Val218 formed favorable interactions with the analyzed ligands. The Cu-A ion showed the highest energetic contribution in the interaction with the KA derivatives. In contrast, Cu-B showed the highest affinity in the interaction with kojic acid.

The analysis of the binding free energy decomposition indicates that the NH_2_, CN, and OH groups present in the KA derivatives formed stable interactions with the residues of the tyrosinase binding pocket. Table S6 shows the average distances computed over the MD simulations between the heavy atoms of the analyzed KA derivatives and the residues of the tyrosinase binding pocket. We noted that the residue Glu195 interacts strongly with the O3 of the KA derivatives (see atoms numbering in Figure S6). The O3 atom improves the affinity to the tyrosinase binding pocket, also forming interactions with residues His60 and His208 that coordinate with the copper ion. Interestingly, Glu195 showed a high energetic contribution with all analyzed KA derivatives (Figure 6).

The NH_2_ group of the KA derivatives also confers high stability to the interaction with the tyrosinase binding pocket. This group formed hydrogen bonds with the residues Met215, Gly216, and Val217 (Figure 6). The Arg209 contains a positively charged group that interacts with the N2 and N1 atoms. It is interesting to note that the derivatives D1 to D6 formed more intermolecular interactions to the binding pocket than the KA molecule, and these additional interactions improve their stability in the complex formation. We also noted that the analyzed KA derivatives with the N1 atom (see Figure S6) interact with the residues Gly216, Val217, and Met215. In addition, the O3 atom formed H-bonds with the residues Glu195 and Asn205, which explains their low binding free energy with the binding pocket (Figure 6). In contrast, the halogens located at the *para* position of the benzene ring of the D1 and D3 did not stabilize the interaction with the residue Phe197. On the other hand, the oxygen present in the *ortho* (D5), *meta* (D6), and *para* (D2 e D4) positions in the benzene ring reduced the repulsion with the residue Phe197, thus reducing the binding free energy. Interestingly, the residues Arg191, His60, Val218, Phe197, and Glu195 showed the highest energetic contributions, which corroborates with previous results that analyzed the tyrosinase inhibition [17,18,19].

## 4. Conclusions

In the present work, we evaluated the binding affinity, selectivity, and structural stability of KA derivatives complexed with tyrosinase, to investigate their structures as potential competitive inhibitors of the melanogenesis in skin cancer. Based on molecular docking analyses, it was possible to verify that the investigated KA derivatives showed favorable interactions with the active site of the enzyme and favorable distance to chelate the Cu^2+^ metals. In comparison with KA, we noted that new interactions were formed with the residues Glu195, Met215, and Asn205. We noted that the copper ions complexed to the active site were well described by the dummy atoms, stabilizing the interactions with the histidine residues over MD simulations. Using binding free energy calculations, we noted that the KA derivatives showed a satisfactory affinity against the tyrosinase active site and they also showed drug-like properties, which are consistent with the cheminformatics filters of Lipinski, Veber, and Muegge. The analysis of pairwise energy decomposition of residue interactions showed that some residues of the L-tyrosine binding site, such as Leu194, Asn205, Val218, and Leu290 contributed energetically to the formation of the inhibitor–receptor complex. Our computational results indicated that the D2, D4, D5, and D6 derivatives are potent inhibitors of tyrosinase activity.

## Figures and Tables

**Table 1:** Docking scores of the natural substrates (L-DOPA and L-tyrosine) and the inhibitors (KA and the fourteen KA derivatives named D1–D14) of tyrosinase.

Type	Compounds	GoldScore
Cofactors	L-DOPA	48.31
	L-tyrosine	48.38
Inhibitors	Kojic acid (KA)	34.98
	D1	43.81
	D2	42.24
	D3	44.45
	D4	43.48
	D5	43.52
	D6	53.20
	D7	50.26
	D8	41.91
	D9	42.84
	D10	53.29
	D11	52.73
	D12	46.08
	D13	43.51
	D14	44.81

**Table 2:** The average distance (Å) obtained from MD simulations between the carbonyl (O1) 4H-pyrone group of the KA and their derivatives with copper ions (Cu-A and -B).

Ligands		D1	D2	D3	D4	D5	D6	KA
**Average distance (Å)**	Cu-A	4.46 (± 0.6)	3.27 (± 0.6)	3.94 (± 0.7)	3.96 (± 1.0)	2.77 (± 0.9)	2.14 (± 0.2)	5.78 (± 0.7)
	Cu-B	6.12 (± 0.4)	5.69 (± 1.0)	6.27 (± 0.7)	5.95 (± 0.6)	5.00 (± 0.3)	5.66 (± 0.4)	4.65 (± 0.4)

**Table 3:** Binding free energies between natural substrates, KA, and its derivatives and the residues of tyrosinase binding site.

Ligands	$V_{l-s}^{vdW}\;bind$	$V_{l-s}^{vdW}\;free$	$V_{l-s}^{el}\;bind$	$V_{l-s}^{el}\;free$	$∆G^{o}\;(\mathrm{kcal}/\mathrm{mol})$
D1	−36.93	−20.15	−50.81	−44.58	−5.09
D2	−90.91	−46.27	−40.81	−22.41	−18.07
D3	−39.06	−21.76	−48.00	−44.10	−4.42
D4	−37.12	−18.68	−78.25	−57.11	−10.31
D5	−98.00	−54.04	−38.86	−18.86	−18.13
D6	−92.73	−49.36	−47.53	−29.11	−17.65
KA	−34.82	−26.22	−22.09	−10.17	−5.00
L-DOPA	−32.74	−13.07	−61.95	−44.54	−12.84
L- tyrosine	−26.62	−13.47	−67.43	−41.58	−9.04

## Data Availability

All data of these manuscript are available online in the supplementary information.

## Supplementary Materials

Table S1: Kojic acid derivatives identified by their ID (D1 to D14), interatomic distance to copper, molecular structure, and IUPAC name. Table S2: Parameters applied to guide the molecular docking simulations of the natural substrates (L-DOPA and L-tyrosinase), kojic acid and its derivatives (cavity radius = 12.193, spatial coordinates of the center of the cavity: x = 1.93, y = 101.58, z = 25.27). Table S3: Average interatomic distances between the carbonyl (O1) 4H-pyrone group of the KA and the derivatives with coppers A and B obtained from MD simulation. Table S4: Average interatomic distances between the histidine residues from the active site and the KA derivatives obtained from MD simulations. Figure S1: RMSD plot of KA and its derivatives complexed with tyrosinase obtained over 5 ns of MD simulation. Figure S2: RMSD plot of the natural substrates (L-tyrosine and L-DOPA) obtained over 5 ns of MD simulation. Figure S3: Energy decomposition of the natural substrate L-DOPA complexed with the tyrosinase binding site. Figure S4: Energy decomposition of the natural substrate L-tyrosine complexed with the tyrosinase binding site. Figure S5: Binding free energy decomposition per residue of tyrosinase complexed with the kojic acid. Table S5: Structural and physicochemical properties calculated for kojic acid and its derivatives. Table S6: Average distance (Å, standard deviation in parenthesis) of the hydrogen bond interactions computed over the MD simulation for the analyzed KA and its derivatives (D1 to D6).
