# Supplementary material for: Analysis of Kojic Acid Derivatives as Competitive Inhibitors of Tyrosinase: A Molecular Modeling Approach
Source: Molecules. 2021 May 12;26(10):2875. doi: 10.3390/molecules26102875 (PMC8152073; doi:10.3390/molecules26102875)
Supplement: Supplementary file 1 [file molecules-26-02875-s001.zip › molecules-1129314-supplementary.pdf]

Supporting Information

Analysis of Kojic Acid Derivatives as Competitive Inhibitors of the Tyrosinase: a Molecular Modeling Approach

Richelly Cardoso [1,3], Renan Valente [2], Clauber Henrique Souza da Costa [3], João Lidio da S. Gonçalves Vianez Júnior [4], Kauê Santana da Costa [3,5,*], Fábio Alberto deMolfetta [1] *and* Cláudio Nahum Alves [3,*]

1 Laboratório de Modelagem Molecular, Instituto de Ciências Exatas e Naturais, Universidade Federal do Pará – UFPA, Guamá, Belém - PA, 66075-10, Brazil
2 Laboratório de Sistemas Moleculares Complexos, Instituto de Ciências Exatas e Naturais, Universidade Federal do Pará – UFPA, Guamá, Belém - PA, 66075-10, Brazil
3 Laboratório de Planejamento e Desenvolvimento de Fármacos. Instituto de Ciências Exatas e Naturais, Universidade Federal do Pará – UFPA, Guamá, Belém - PA, 66075-10, Brazil
4 Center of Technological Innovation, Evandro Chagas Institute, Ministry of Health, Ananindeua, PA, 67030-000, Brazil
5 Universidade Federal do Oeste do Pará, Instituto de Biodiversidade, 68035-110, Santarém, PA Brazil

Table S1. Kojic acid derivatives identified by their ID (D1 to D14), interatomic distance to copper, molecular structure, and IUPAC name.

Molecule ID	Interatomic distance to copper (Å)	Molecular structure	IUPAC name
D1	C=O .. Cu^{+2} (3.762)	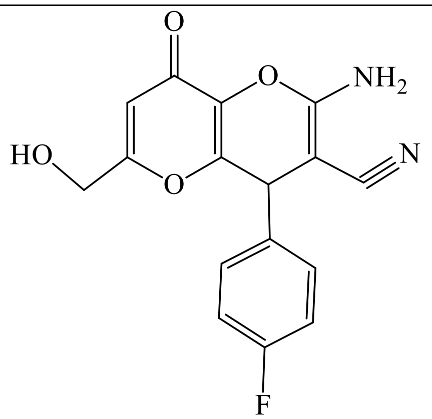	2-amino-4-(4-fluorophenyl)-6-(hydroxymethyl)-8-oxo-4H,8H-pyrano[3,2-b]pyran-3-carbonitrile
D2	C=O .. Cu^{+2} (3.912)	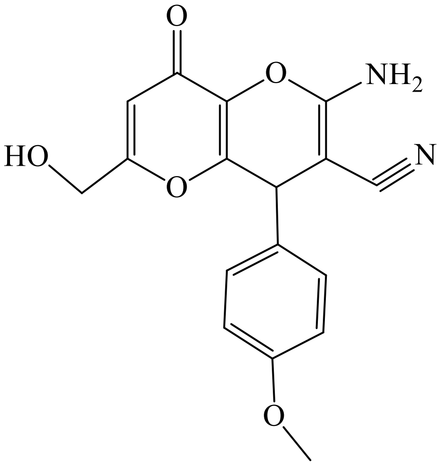	2-amino-6-(hydroxymethyl)-4-(4-methoxyphenyl)-8-oxo-4H,8H-pyrano[3,2-b]pyran-3-carbonitrile

D3	C=O .. Cu^{+2} (3.693)	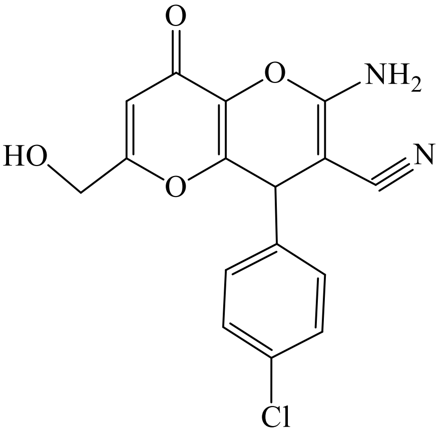	2-amino-4-(4-chlorophenyl)-6-(hydroxymethyl)-8-oxo-4H,8H-pyrano[3,2-b]pyran-3-carbonitrile
D4	C=O .. Cu^{+2} (3.642)	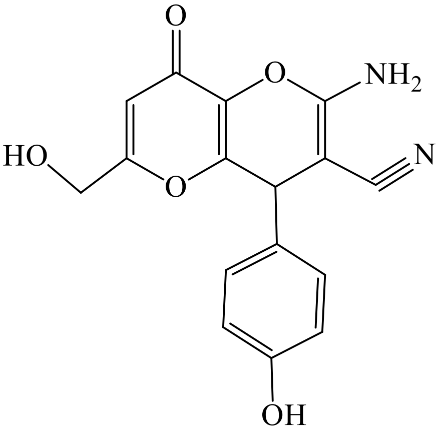	2-amino-6-(hydroxymethyl)-4-(4-hydroxyphenyl)-8-oxo-4H,8H-pyrano[3,2-b]pyran-3-carbonitrile
D5	C=O .. Cu^{+2} (3.715)	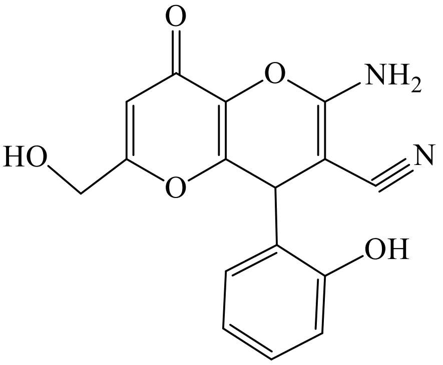	2-amino-6-(hydroxymethyl)-4-(2-hydroxyphenyl)-8-oxo-4H,8H-pyrano[3,2-b]pyran-3-carbonitrile
D6	C=O .. Cu^{+2} (3.667)	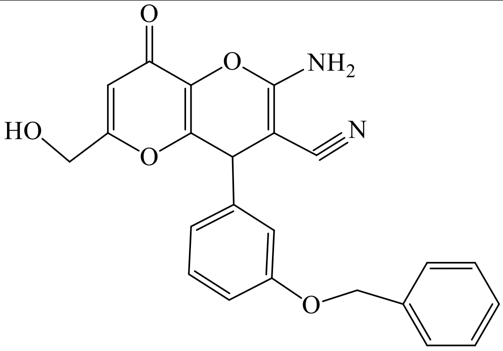	2-amino-4-[3-(benzyloxy)phenyl]-6-(hydroxymethyl)-8-oxo-4H,8H-pyrano[3,2-b]pyran-3-carbonitrile
D7	C=O .. Cu^{+2} (12.213)	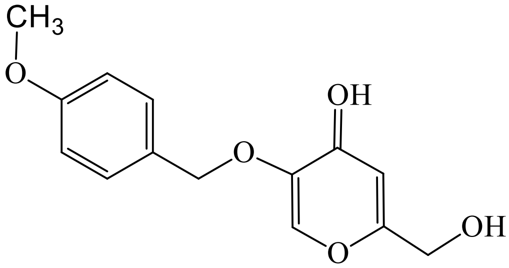	2-(hydroxymethyl)-5-[(4-methoxyphenyl)methoxy]-4H-pyran-4-one

D8	C=O .. Cu^{+2} (7.040)	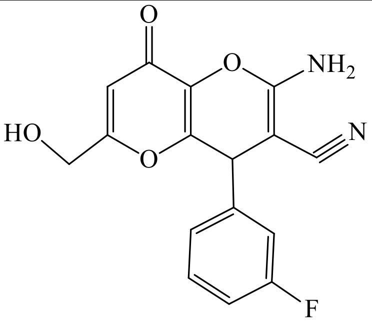	2-amino-4-(3-fluorophenyl)-6-(hydroxymethyl)-8-oxo-4H,8H-pyrano[3,2-b]pyran-3-carbonitrile
D9	C=O .. Cu^{+2} (3.858)	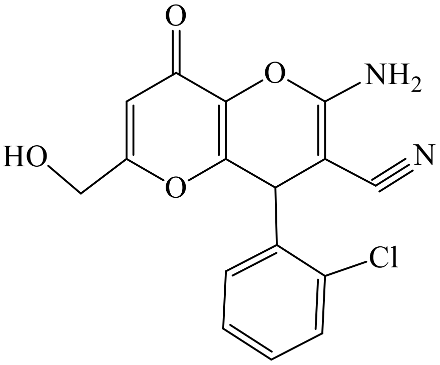	2-amino-4-(2-chlorophenyl)-6-(hydroxymethyl)-8-oxo-4H,8H-pyrano[3,2-b]pyran-3-carbonitrile
D10	C=O .. Cu^{+2} (8.144)	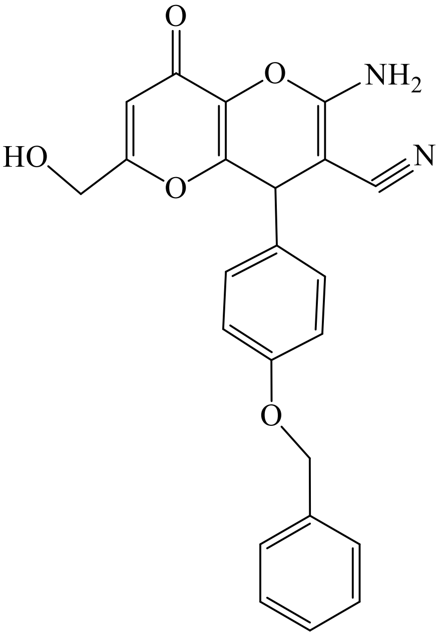	2-amino-4-[4-(benzyloxy)phenyl]-6-(hydroxymethyl)-8-oxo-4H,8H-pyrano[3,2-b]pyran-3-carbonitrile
D11	C=O .. Cu^{+2} (17.008)	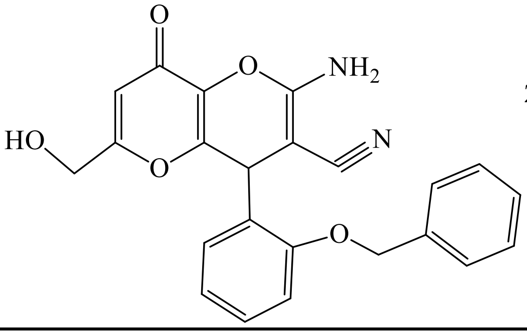	2-amino-4-[2(benzyloxy)phenyl]-6-(hydroxymethyl)-8-oxo-4H,8H-pyrano[3,2-b]pyran-3-carbonitrile

D12	C=O .. Cu^{+2} (3.667)	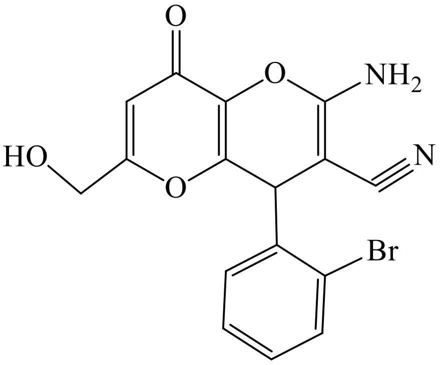	2-amino-4-(2-bromophenyl)-6-(hydroxymethyl)-8-oxo-4H,8H-pyrano[3,2-b]pyran-3-carbonitrile
D13	C=O .. Cu^{+2} (3.771)	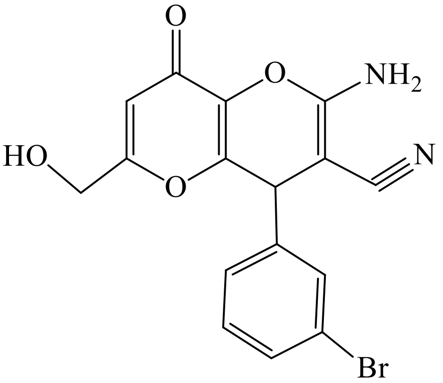	2-amino-4-(3-bromophenyl)-6-(hydroxymethyl)-8-oxo-4H,8H-pyrano[3,2-b]pyran-3-carbonitrile
D14	C=O .. Cu^{+2} (11.516)	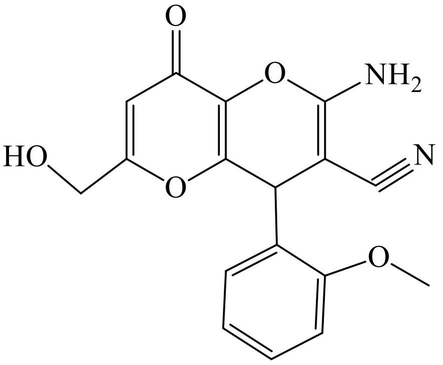	2-amino-6-(hydroxymethyl)-4-(2-methoxyphenyl)-8-oxo-4H,8H-pyrano[3,2-b]pyran-3-carbonitrile

Table S2. Parameters applied to guide the molecular docking simulations of the natural substrates (L-DOPA and L-tyrosinase), kojic acid and its derivatives (cavity radius = 12.193, spatial coordinates of the center of the cavity: x = 1.93, y = 101.58, z = 25.27).

Parameters	D1	D2	D3	D4	D5	D6	KA	L-DOPA	L-Tyrosine
n-ops	35250	42000	35250	42000	42000	55250	28000	44000	35500
n-islands	3	4	3	4	4	5	2	4	3
Population size	100	100	100	100	100	100	100	100	100
mutation	95	95	95	95	95	95	95	95	95
crossover	95	95	95	95	95	95	95	95	95
migration	10	10	10	10	10	10	10	10	10
pressure	1.100	1.100	1.100	1.100	1.100	1.100	1.100	1.100	1.100

Table S3. Average interatomic distances between the carbonyl (O1) 4H-pyrone group of the KA and the derivatives with coppers A and B obtained from MD simulation.

Ligand		D1	D2	D3	D4	D5	D6	KA
**Average distance** (Å)	Cu-A	4.250	4.240	3.693	4.073	4.011	4.070	4.790
	Cu-B	3.762	3.912	2.832	3.642	3.715	3.667	5.534

Table S4. Average interatomic distances between the histidine residues from the active site and the KA derivatives obtained from MD simulations.

KA derivative D1	
**Histidine/Cu-A**	**Average distance interaction**

His200 (NE2)	2.325 ± 0.08
His204 (NE2)	2.394 ± 0.11
His227 (NE2)	2.164 ± 0.04
**Histidine/Cu-B**	**Average distance interaction**
His38 (NE2)	2.165 ± 0.04
His56 (NE2)	2.309 ± 0.08
His65 (NE2)	2.166 ± 0.04
**KA derivative D2**	
**Histidine/Cu-A**	**Average distance interaction**
His200 (NE2)	2.259 ± 0.08
His204 (NE2)	2.137 ± 0.04
His227 (NE2)	2.152 ± 0.05
**Histidine/Cu-B**	**Average distance interaction**
His38 (NE2)	2.235 ± 0.09
His56 (NE2)	2.235 ± 0.10
His65 (NE2)	2.181 ± 0.05
**KA derivative D3**	
**Histidine/Cu-A**	**Average distance interaction**
His200 (NE2)	2.238 ± 0.07
His204 (NE2)	2.140 ± 0.04
His227 (NE2)	2.162 ± 0.06
**Histidine/Cu-B**	**Average distance interaction**
His38 (NE2)	2.245 ± 0.09
His56 (NE2)	2.206 ± 0.07
His65 (NE2)	2.171 ± 0.04
**KA derivative D4**	
**Histidine/Cu-A**	**Average distance interaction**
His200 (NE2)	2.261 ± 0.08
His204 (NE2)	2.127 ± 0.04
His227 (NE2)	2.149 ± 0.04
**Histidine/Cu-B**	**Average distance interaction**
His38 (NE2)	2.169 ± 0.04
His56 (NE2)	2.281 ± 0.11
His65 (NE2)	3.176 ± 0.65
**KA derivative D5**	
**Histidine/Cu-A**	**Average distance interaction**
His200 (NE2)	2.273 ± 0.10
His204 (NE2)	2.138 ± 0.04
His227 (NE2)	2.151 ± 0.20
**Histidine/Cu-B**	**Average distance interaction**
His38 (NE2)	2.152 ± 0.04
His56 (NE2)	2.160 ± 0.04
His65 (NE2)	2.156 ± 0.06
**KA derivative D6**	
**Histidine/Cu-A**	**Average distance interaction**
His200 (NE2)	2.162 ± 0.06
His204 (NE2)	2.141 ± 0.04
His227 (NE2)	2.295 ± 0.14
**Histidine/Cu-B**	**Average distance interaction**
His38 (NE2)	2.280 ± 0.17
His56 (NE2)	2.246 ± 0.10
His65 (NE2)	2.179 ± 0.05
**Kojic acid**	
**Histidine/Cu-A**	**Average distance interaction**
His200 (NE2)	2.383 ± 0.11
His204 (NE2)	2.185 ± 0.04
His227 (NE2)	2.197 ± 0.08
**Histidine/Cu-B**	**Average distance interaction**
His38 (NE2)	2.233 ± 0.08

His56 (NE2)	2.218 ± 0.13
His65 (NE2)	3.242 ± 0.62

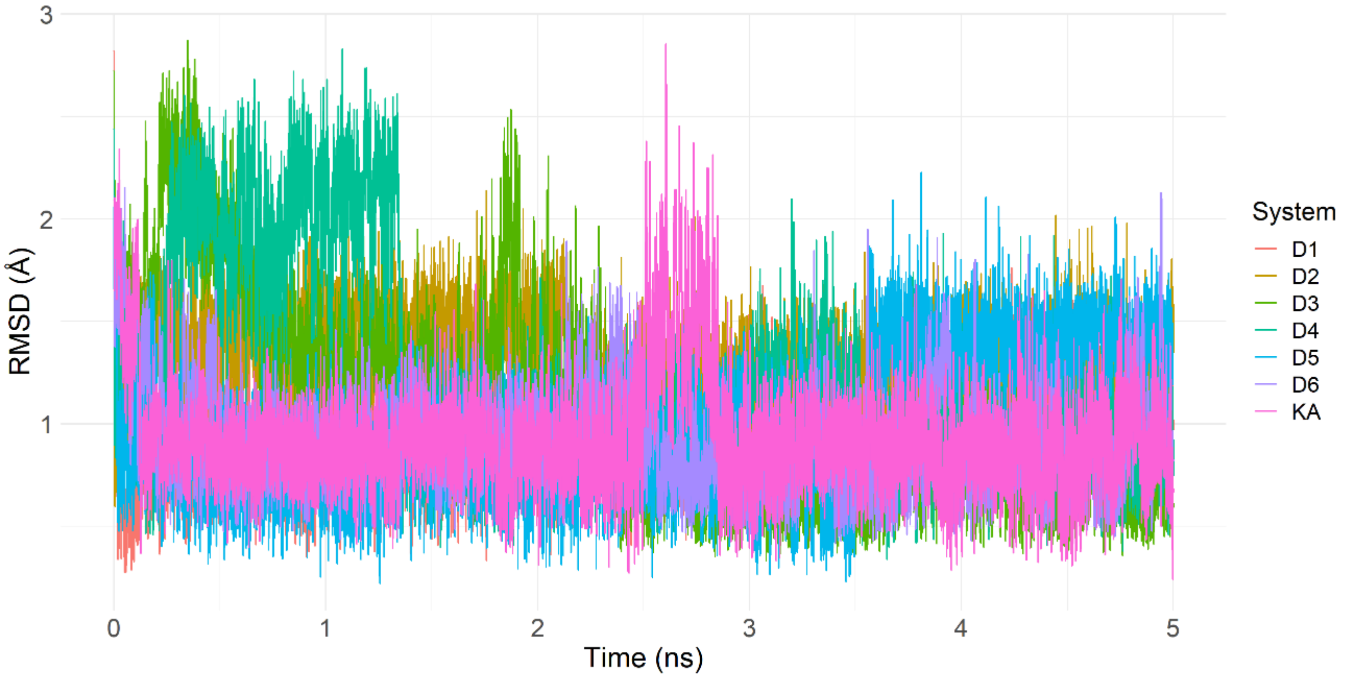

Figure S1. RMSD plot of KA and its derivatives complexed with tyrosinase obtained over 5ns of MD simulation.

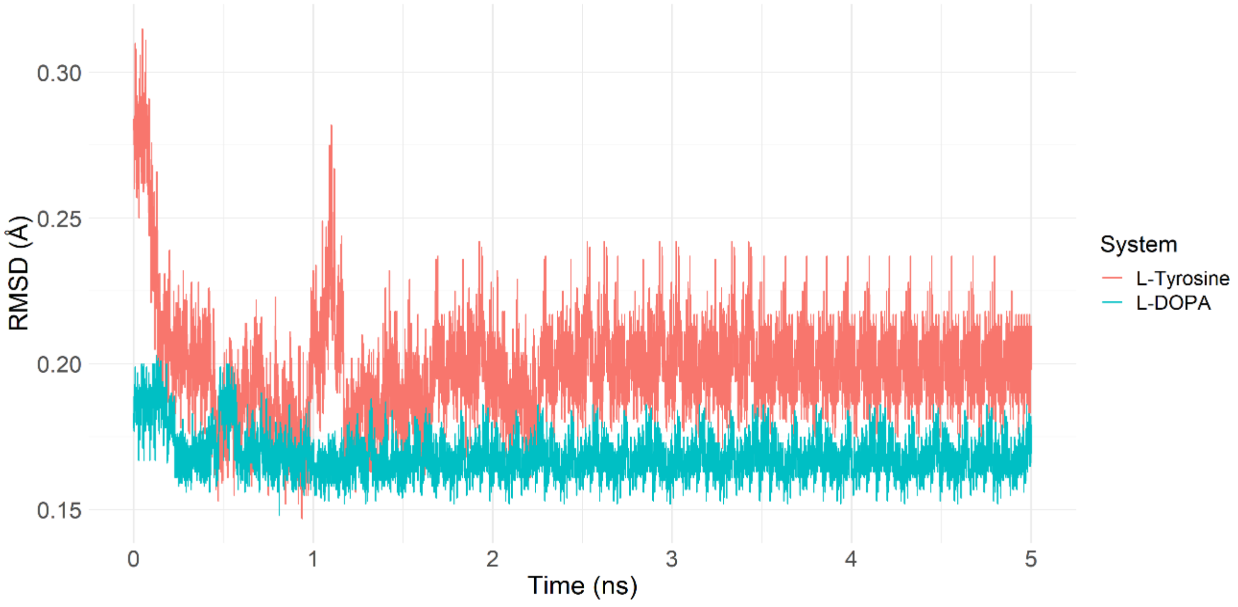

Figure S2. RMSD plot of the natural substrates (L-tyrosine and L-DOPA) obtained over 5ns of MD simulation.

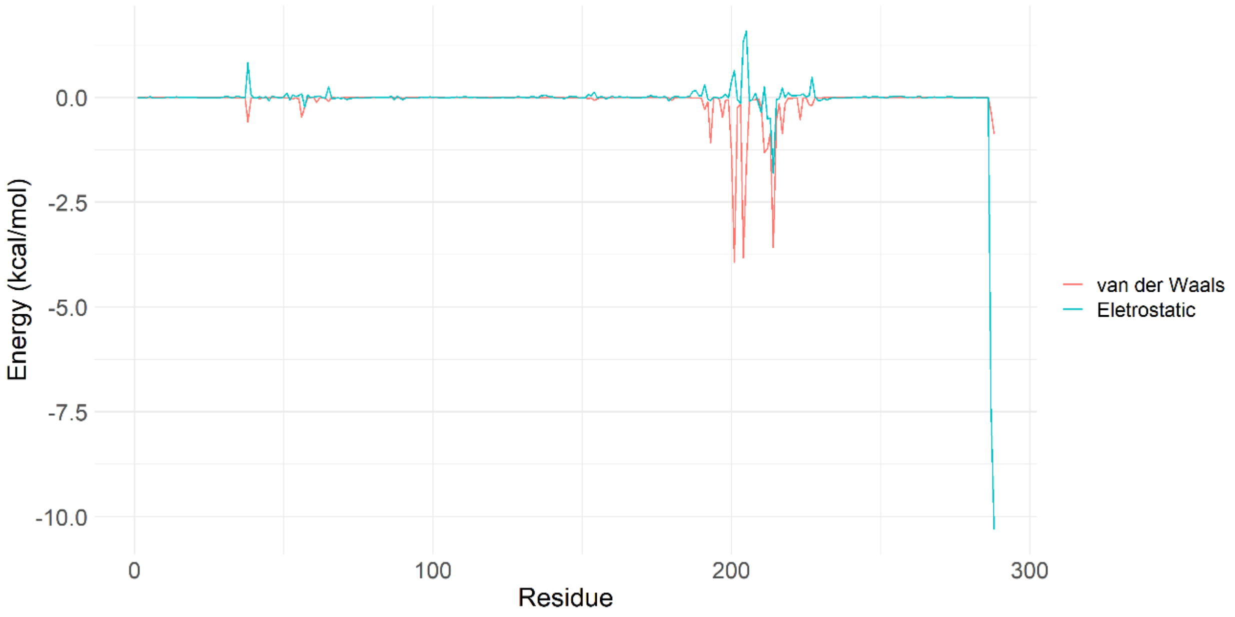

Figure S3. Energy decomposition of the natural substrate L-DOPA complexed with the tyrosinase binding site.

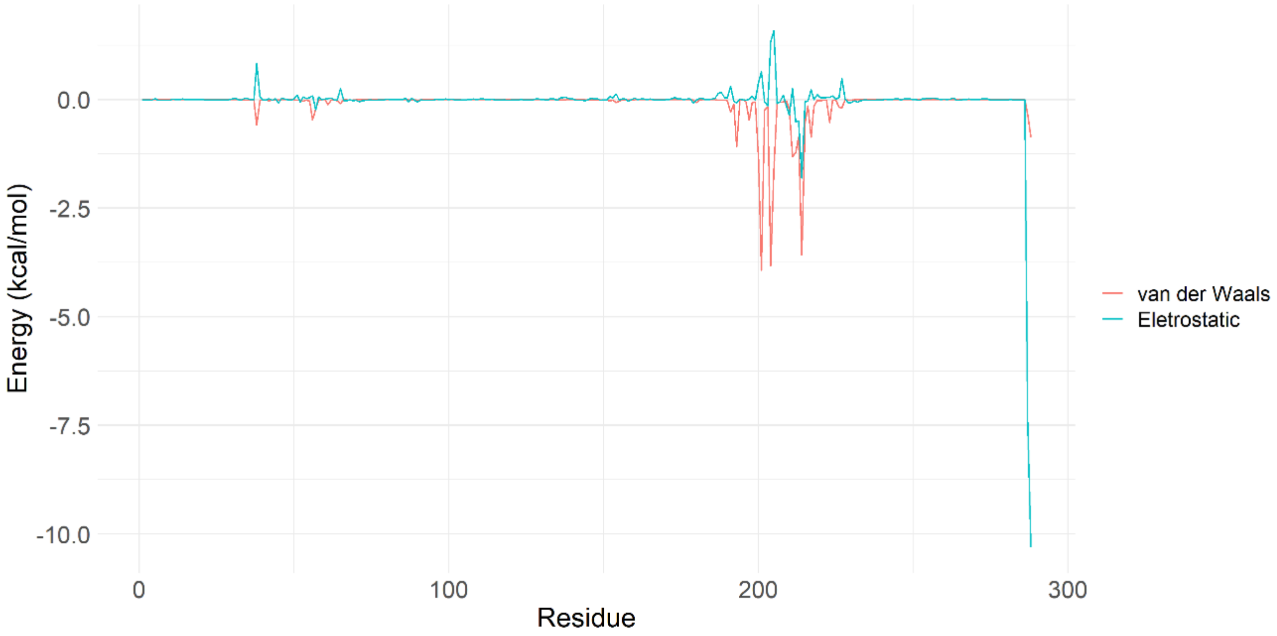

Figure S4. Energy decomposition of the natural substrate L-tyrosine complexed with the tyrosinase binding site.

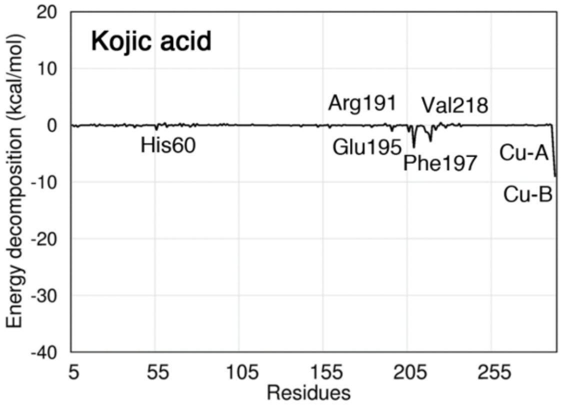

Figure S5. Binding free energy decomposition per residue of tyrosinase complexed with the kojic acid.

Table S5. Structural and physicochemical properties calculated for kojic acid and its derivatives.

CdId	Structure	Mol Weight	Formula	Name	LogD	LogP	Molecular weight	TPSA	H bond acceptors	H bond donors	Atom count	Rotatable bonds	Ring count	Lipinski (4 of 4)	Muegge	Veber
1	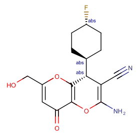	320,32	C16H17FN2O4	D1	0,646	0,646	320,32	105,57	6	2	40	2	3	true	true	true
2	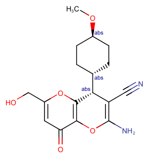	332,356	C17H20N2O5	D2	0,398	0,398	332,356	114,8	7	2	44	3	3	true	true	true
3	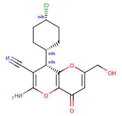	336,77	C16H17ClN2O4	D3	1,110	1,110	336,77	105,57	6	2	40	2	3	true	true	true
4	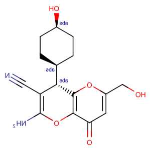	318,329	C16H18N2O5	D4	0	0	318,329	125,8	7	3	41	2	3	true	true	true
5	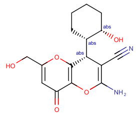	318,329	C16H18N2O5	D5	0	0	318,329	125,8	7	3	41	2	3	true	true	true
6	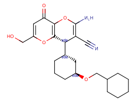	414,502	C23H30N2O5	D6	2,513	2,513	414,502	114,8	7	2	60	5	4	true	true	true

Table S6. Average distance (Å, standard deviation in parenthesis) of the hydrogen bond interactions computed over the MD simulation for the analyzed KA and its derivatives (D1 to D6).

Inhibitor atoms D1	Residues	Distance	Inhibitor atoms D2	Residues	Distance
O2	Asn205-ND2	3.58 (0.41)	O3	Glu195-OE2	2.63 (0.11)
O3	Glu195-OE2	2.63 (0.10)	N1	Gly216-O	2.87 (0.28)
	Asn205-ND2	5.04 (0.56)		Val217-O	4.75 (1.44)
	His208-ND1	3.54 (0.37)		Met215-O	4.17 (1.03)
N1	Met215-O	4.86 (0.67)	N2	Arg209-NH2	5.65 (1.04)
	Val217-O	5.01 (1.16)			
	Val218-N	4.64 (1.06)			
	Gly216-O	2.97 (0.28)			
N2	Arg209-NH1	5.04 (1.01)			
**D3**	**Residues**	**Distance**	**D4**	**Residues**	**Distance**
O3	His60-ND1	4.23 (0.62)	O3	Glu195-OE2	2.59 (0.09)
	Met61-SD	4.88 (0.73)		Asn205-ND2	5.24 (0.68)
	Glu195-OE2	2.92 (0.52)	N1	Arg209-NE	5.41 (1.37)
	Asn205-ND2	5.87 (0.81)		Arg209-NH1	6.08 (1.21)
N1	His208-ND1	4.31 (0.72)		Met215-O	4.49 (1.00)
	Arg209-NH1	5.78 (0.98)		Gly216-O	3.04 (0.65)
	Met215-O	3.46 (0.85)		Val217-O	4.80 (1.25)
	Gly216-O	2.91 (0.32)	N2	Arg209-NH1	5.05 (0.72)
	Val217-O	3.95 (1.14)		Arg209-NE	4.02 (0.52)
N2	Arg209-NH1	4.24 (0.81)			
	Arg209-NE	5.46 (0.88)			
**D5**	**Residues**	**Distance**	**D6**	**Residues**	**Distance**
O2	Asn205-ND2	5.41 (0.43)	O3	Met61-N	3.77 (0.38)
O3	Glu195-OE2	2.61 (0.09)		Glu195-OE2	2.54 (0.08)
O5	Asn205-ND2	3.85 (0.50)	N1	His208-ND1	3.33 (0.26)
N1	Met215-O	3.12 (0.29)		Met215-O	3.05 (0.21)
	Gly216-O	3.19 (0.78)		Gly216-O	2.92 (0.34)
	Val217-O	3.14 (0.37)		Val217-O	3.74 (0.78)
N2	Arg209-NH1	4.90 (0.75)	N2	Arg209-NH1	5.38 (0.71)
**KA**	**Residues**	**Distance**			
O1	Asn205-OD1	5.33 (1.17)			
	Asn205-ND2	6.17 (1.03)			
	Arg209-NH1	5.87 (0.87)			
	Arg209-NE	5.188 (0.69)			
	Met215-O	4.53 (1.02)			
	Gly216-O	4.08 (0.59)			
	Val217-O	5.82 (1.14)			
O4	Asn205-OD1	5.40 (0.96)			

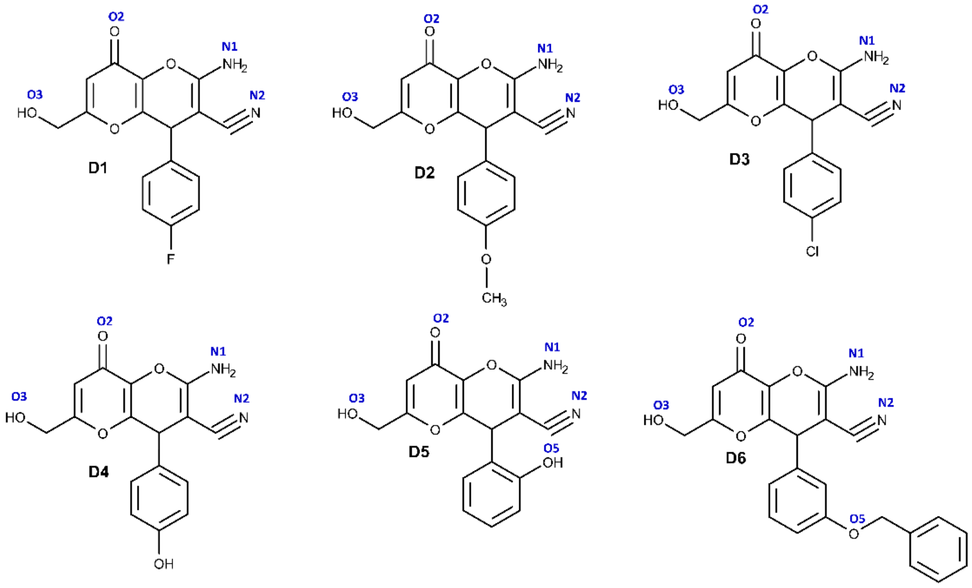

Figure S6. Heavy atoms numbering of the KA derivatives (D1 to D6).
